# Quantifying the relationship between specialisation and reputation in an online platform

**DOI:** 10.1038/s41598-022-20767-7

**Published:** 2022-10-06

**Authors:** Giacomo Livan, Giuseppe Pappalardo, Rosario N. Mantegna

**Affiliations:** 1 Department of Computer Science, University College London, Gower Street, London, WC1E 6EA UK; 2 Systemic Risk Centre, London School of Economics and Political Sciences, Houghton Street, London, WC2A 2AE UK; 3 Dipartimento di Fisica ‘Ettore Majorana’, Università di Catania, Via S. Sofia, 64, 95123 Catania, Italy; 4 Dipartimento di Fisica e Chimica Emilio Segrè, Università di Palermo, Viale delle Science, Ed. 18, 90128 Palermo, Italy; 5 Complexity Science Hub Vienna, Josefstädter Strasse 39, 1080 Vienna, Austria

**Keywords:** Computational science, Complex networks

## Abstract

Online platforms implement digital reputation systems in order to steer individual user behaviour towards outcomes that are deemed desirable on a collective level. At the same time, most online platforms are highly decentralised environments, leaving their users plenty of room to pursue different strategies and diversify behaviour. We provide a statistical characterisation of the user behaviour emerging from the interplay of such competing forces in Stack Overflow, a long-standing knowledge sharing platform. Over the 11 years covered by our analysis, we represent the interactions between users and topics as bipartite networks. We find such networks to display nested structures akin to those observed in ecological systems, demonstrating that the platform’s user base consistently self-organises into specialists and generalists, i.e., users who focus on narrow and broad sets of topics, respectively. We relate the emergence of these behaviours to the platform’s reputation system with a series of data-driven models, and find specialisation to be statistically associated with a higher ability to post the best answers to a question. We contrast our findings with observations made in top-down environments—such as firms and corporations—where generalist skills are consistently found to be more successful.

## Introduction

The evolution of the digital economy has transformed several top-down online environments into bottom-up, decentralised platforms. For instance, information and news are now largely consumed via social media, and well established business-to-consumer sectors—such as the hotel industry^1^—have been disrupted by sharing economy platforms, which empower users to become small entrepreneurs by sharing spare resources.

Because of their decentralised nature, over the years several online platforms have introduced a variety of incentive systems to foster trust between their users. In some cases (e.g., Twitter), these come as simple identity verification protocols. In other cases (e.g., sharing economy platforms such as Uber and Airbnb), trust is fostered with a reputation score that users develop through digital peer-review mechanisms (e.g., star ratings)^2,3^.

Online reputation systems effectively reward/punish specific actions, ultimately selecting which kinds of user behaviours—and therefore which users—may get to experience sustained success in a platform. Depending on a platform’s nature, this may translate into substantial economic gains. For instance, plenty of both anecdotal and direct evidence shows that high reputation scores on Stack Overflow—the platform we will use as data source in this study—correlate with improved employment prospects for programmers and developers^4^.

The extensive game-theoretic literature on the evolution of cooperation has explored—both theoretically and experimentally—how individuals can establish a reputation in a variety of social contexts^5^, focusing, e.g., on explanatory mechanisms grounded in indirect reciprocity^6–8^ or in the conditional response to the behaviour of others^9^. In recent years, the link between user behaviour and reputation has been analysed by a number of studies in the novel context of online platforms. Experimental approaches have measured user response to different elements appearing on profiles in order to identify which ones are most conducive to trust^10,11^. Other studies have instead looked at strategic behaviour as a driver of user reputation, focusing, e.g., on the cooperative and retaliatory mechanisms underlying the exchange of ratings^12,13^ and on the incentives to commit review fraud^14^. An understudied aspect in this stream of research relates to other types of strategic user behaviours, namely those related to specialisation and/or generalism.

In ecology, the term specialist (generalist) refers to species that prosper in a limited (wide) range of environmental conditions. Specialisation emerges as a natural response to competitive pressure, with the aim of securing an edge in specific circumstances. Conversely, generalism emerges as resilience against varying conditions. Such concepts have found plenty of applications in non-natural domains, and have been particularly helpful to conceptualise different strategic behaviours in large socio-economic systems.

The management literature has consistently found that individuals with broader sets of skills (i.e., generalists) enjoy greater success in top-down organisations. Generalist CEOs receive higher pay than their specialist counterparts, with the highest pay increases occurring when firms switch from a specialist to a generalist CEO^15^. Similar results are found in^16^, which the authors interpreted as a reflection of a higher demand for generalist skills required to manage increasingly complex firms, and generalist CEOs are more likely to engage in acquisitions outside a firm’s main industry^17^. Similarly, empirically tested theories of leadership support the idea that leaders in industry tend to be generalists rather than specialists^18^.

Traces of such behaviours—ranging from extreme specialisation to extreme generalism—have been also observed in the bottom-up context of online platforms, leading to the identification of sharply distinct user archetypes. For instance, Reddit users are found to follow a variety of ‘wandering’ patterns as they hop across different communities^19^, with specialists eventually being more likely to stick to the few selected online communities they contribute to, and generalists being more likely to remain active on the platform as a whole and to interact with a more diverse set of users^20^. Markedly different user profiles have also been detected in community Q &A platforms. The emergence of users specialised, e.g., in answering questions or commenting on specific posts (e.g., their own vs those of others) has been documented, and the prevalence of such profiles has been found to correlate significantly with the main defining topic of a community (e.g., arts vs sciences) and with its health metrics (e.g., fraction of questions that receive an answer)^21^. In the context of online gaming, generalists have been found to be more resilient to change (e.g., after the release of game patches) although specialists ultimately tend to outperform other players on average^22^. Notably, evidence of specialisation and genearalism in online environments has also been detected in non-human agents^23^.

In this paper, we aim to quantify the relationship between specialisation/generalism and reputation in online platforms. To the best of our knowledge, this is an understudied relationship, which has only been looked at in contexts where reputation is developed through interactions that are external to platforms (e.g., the online ratings received by medical professionals on physician-rating websites^24^). Our focus here, instead, is to look at such a link in contexts where user reputation is developed *endogenously* through interactions and peer-review taking place on the platform itself. We do so by analysing data from Stack Overflow (SO), the flagship knowledge-sharing platform of the Stack Exchange network, which features questions and answers on a wide variety of topics in the area of computer programming (see “Methods” section). SO implements an elaborate reputation system, which is well known for its effectiveness in incentivising users to produce high quality posts^25^.

## Results

The portion of the SO data used in our study spans 11 years going from January 2009 (shortly after the platform was started in 2008) to December 2019. Posts represent the main unit of activity in the platform. Posts are divided into three main categories: questions, answers, and accepted answers. An accepted answer is a post that has been identified as the best one in response to a question by the author of that question. Users can classify the questions they post with up to five tags (e.g., C++, Python, etc), which help other users identify the posts they might be able to reply to. Each individual post (i.e., both questions and answers) can generate a sub-thread in the form of comments. Any post or comment can be either up-voted or down-voted by other users. Users develop a reputation score based on their activity. The main source of points are accepted answers ($+15$ points) and up-votes ($+5$ for questions, $+10$ for answers). A down-vote penalizes the user receiving it by $-2$ points. In line with the literature on punishment and reputation^26^, down-voting posts is made costly ($-1$ point) in order to suppress trolling. Upon reaching certain milestones users can also earn reputational badges (see, e.g.,^27^).

### Platform growth

**Figure 1:**
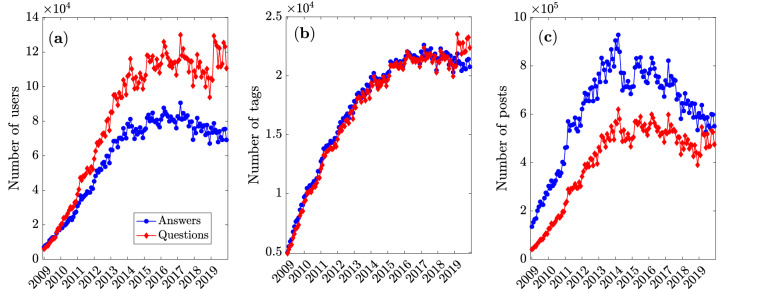
Growth of stack overflow from 2009 to 2019. (**a**) Monthly number of active users. (**b**) Monthly number of tags featured in posts. (**c**) Monthly number of posts. In all panels the blue (red) symbols refer to answers (questions) on Stack Overflow.

We begin our analysis by looking at the evolution of the Stack Overflow platform over time from an aggregate perspective. For each year in our dataset (2009-2019), we look at the monthly number of active users (i.e., users who posted at least once), the monthly number of tags (i.e., tags that appear in at least one post), and the monthly number of posts. These quantities are reported in Fig. 1, broken down by post type. The monthly number of users posting questions rapidly overtakes the monthly number of those posting answers (left panel), and both numbers settle around several thousands during 2013-2014. The number of tags featured in answers and questions roughly equal each other throughout the platform’s lifetime (central panel), whereas the number of answers posted remains systematically higher than the number of questions (right panel), with both numbers settling on the order of hundreds of thousands of posts per month.

We then proceed to characterise the platform’s growth by categorising its user base with respect to post types. We discard casual users by restricting our analysis to those who contribute with at least 10 posts (answers and questions combined) in a given year. We characterise a user’s activity based on the relative proportion of questions and answers. We indicate as $A_i(y)$ ($Q_i(y)$) the number of answers (questions) posted by user *i* during year *y*, and we characterise the user’s profile with respect to post types in that year with the following score:

1 $$\begin{aligned} D_i(y) = \frac{A_i(y)-Q_i(y)}{A_i(y)+Q_i(y)} \ . \end{aligned}$$

**Figure 2:**
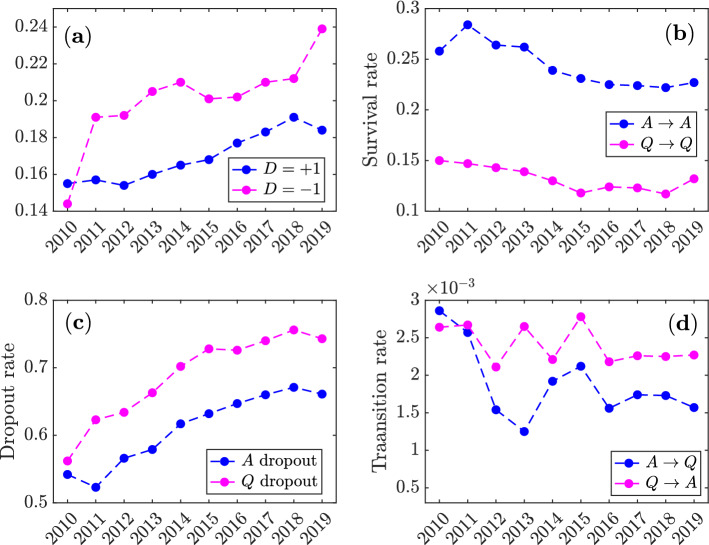
Characterisation of stack overflow’s user base. (**a**) Annual percentage of *A*- and *Q*-users ($D=1$ and $D=-1$, respectively, see Eq. (1)). (**b**) Annual survival probabilities for *A*- (blue) and *Q*-users (magenta), defined as the empirically estimated probabilities for users belonging to either group to belong to the same group in the following year. (**c**) Annual dropout rates for *A*- (blue) and *Q*-users (magenta), defined as the empirically estimated probabilities for users belonging to either group to either leave the platform or fall below the minimum threshold of 10 posts per year to be considered in our analysis. $(\mathbf {d})$ Annual transition rates from *A*- to *Q*-users (blue) and vice versa (magenta), defined as the empirically estimated probabilities for users belonging to one group to transition to the other one the following year.

Figure 2 (top left) shows the annual proportions of users who only post answers ($D_i = +1$) or questions ($D_i = -1$). Let us label such two groups as *A*-users and *Q*-users, respectively. Overall, the proportions of users belonging to both groups grow over time. However, the fraction of *A*-users remains relatively stable between $15\%$ and $20\%$ (even showing some decline in 2019), whereas the proportion of *Q*-users increases from less than $15\%$ to almost $25\%$. After an initial phase where *A*-users are more numerous, *Q*-users become the relative majority in 2011, signalling the platform’s transition from a ‘supply-driven’ to a ‘demand-driven’ knowledge marketplace.

The above transition is not driven by the addition of newcomers to a stable core of users, but rather by turnover. The top right and bottom left panels in Fig. 2 show—respectively—the year-to-year survival and dropout rates for *A*- and *Q*-users. With the former, we indicate the empirically estimated probability that a user belonging to either group in a given year will again belong to the same group the following year, while with the latter we indicate the probability that a user either leaves the platform or falls below the minimum activity threshold to be included in our analysis (10 posts). Only a minority of *A*- and *Q*-users remain in such groups in consecutive years, and the dropout rates for both groups display a sharp increase over time. We can therefore conclude that the sub-populations of *A*- and *Q*-users grow over time through the replacement of users who drop out with larger numbers of new users. Those results are robust to changes in the threshold on the minimum number of posts for users to be included in our analysis (see Supplementary Information S1).

Let us also mention that there is very little spillover between the two groups throughout the years, as testified by the fact that the transition rates between them (i.e., the empirically estimated probability that a *Q*-user will become an *A*-user the following year and vice versa) are both below $0.3\%$, as shown in the bottom right panel in Fig. 2 .

### Specialist and generalist users

**Figure 3:**
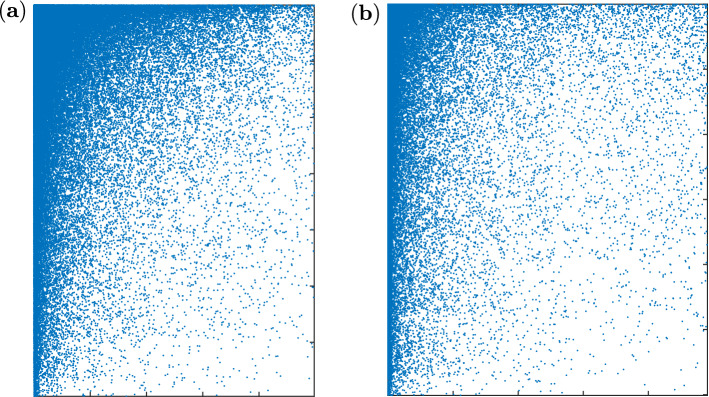
Evidence of nestedness in Stack Overflow’s user-tag bipartite networks. (**a**) User-tag bipartite network for answers posted in January 2009 (size: $6960 \times 4982$). (**b**) User-tag bipartite network for questions posted in January 2009 (size: $6011 \times 4906$). In both panels blue dots represent non-zero entries, and the matrix rows and columns have been sorted from top to bottom for users and from left to right for tags.

We then proceed to characterize user behaviour in terms of topics. We do so by forming weighted monthly bipartite user-tag networks restricted to ‘pure’ *A*- and *Q*- users (i.e., users whose activity score in Eq. (1) is $D = 1$ and $D = -1$, respectively, in the year of interest). Namely, if a *Q*-user *i* has posted $w_{i\tau }^Q$ questions featuring the tag $\tau$, we place a link from *i* to $\tau$ with weight $w_{i\tau }^Q$. We construct a similar network for *A*-users, considering as weights the number of answers posted in response to questions featuring a certain tag. Following well established approaches to detect the coexistence of specialisation and generalism in ecosystems, we measure *nestedness* in such networks (see Fig. 3).

In ecological networks of species-species interactions (e.g., hosts-parasites), nestedness refers to the fact that the species with which specialists interact are a subset of those with which generalists interact (a property which becomes visually clear when sorting the rows and columns of the networks’ adjacency matrix based on node degree, as done in Fig. 3). Notably, networks with heterogeneous degree distributions often display spurious nestedness. In this case, the statistical significance of the nestedness measured in empirical networks must be tested against values of nestedness obtained under a suitable null network model. Evaluating nestedness and its significance in weighted networks—such as those that we consider in this analysis—is further complicated by the distribution of weights on links, which may act as a further confounder. In this case, methods to quantify nestedness often rely on generalising concepts that apply to the unweighted case. We adopt a similar approach and follow a procedure based on spectral radii^28^. In a nutshell, in the unweighted case perfectly nested matrices are those that maximise the spectral radius of an adjacency matrix. This property can be leveraged to detect statistically significant nestedness in weighted adjacency matrices by measuring their spectral radius and how it deviates from its expected value in a suitable null model (see “Methods” section). With this method, we find nestedness to be statistically significant throughout the platform’s history (see Supplementary Information S1), which in turn suggests that the platform indeed self-organises into specialist and generalist users, both in its supply and demand sides.

Based on this observation, we then quantify the level of specialisation attained by users in their activity when posting answers/questions with the Herfindahl index, a measure of concentration which (in the case of questions) reads

2 $$\begin{aligned} H_i^Q = \sum _\tau \left( \frac{w_{i\tau }^Q}{s_i^Q} \right) ^2 \ , \end{aligned}$$

where $w_{i\tau }^Q$ (as defined above) is the number of questions posted by the user on tag $\tau$, whereas $s_i ^Q= \sum _\tau w_{i\tau }^Q$ is the total number of questions posted by the user. With the above definition, the Herfindahl index will approach one for users who are only active on a limited set of tags (with the limiting case $H_i^Q = 1$ for users active on just one tag), and will instead approach zero for users whose activity is uniformly spread over a large number of tags. We define an equivalent index $H_i^A$ in the case of answers and characterise users whose activity features both types of posts with both Herfindahl indices.

In the Supplementary Information S1, we show the annual distributions of the Herfindahl scores for both answers and questions. Both distributions are remarkably stable throughout the years, signalling that—despite the increase in the number of tags (see the middle panel in Fig. 1)—the users’ collective behaviour in terms of specialization remains largely unchanged.

### Reputation

We proceed next to investigate the users’ reputation in the platform. For each user with at least 10 posts in a year, we build a profile based on the following features describing their activity: the number of posts (*n*), the number of tags associated to their posts (*t*), their Herfindahl indices ($H^A$ and $H^Q$, see Eq. (2)), and their activity score (*D*, see Eq. (1)). We use these features to build a number of linear models to characterise user reputation on the platform.

**Figure 4:**
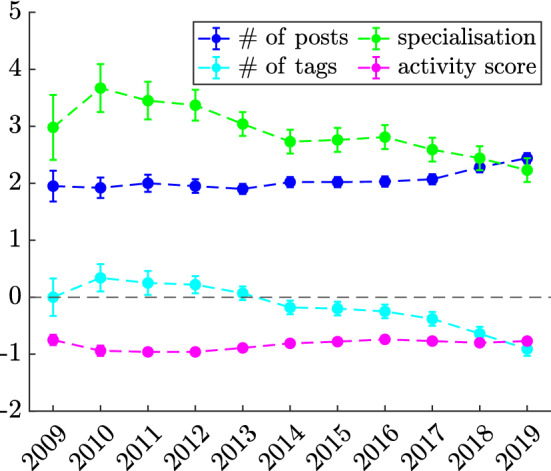
Logistic regression results for the probability that a user has at least one accepted answer in a given year. Dots represent the values of the regression coefficients estimated for the four covariates included in the model, shown in the legend. Error bars show the standard errors on the coefficients times three.

We begin by looking at the main sources of user reputation, i.e., the ability to post *accepted* answers. These correspond to answers selected as the best one in response to a given question by the author of the very same question. Notably, posting an accepted answer is worth 15 reputation points (whereas an up-vote, for instance, is worth 10), and it is the result of a combination of skills (i.e., both competence and rapidity). In order to identify the factors that are conducive to a user’s ability to post answers that may get accepted, we consider a logistic regression model for the log-odds $\log (\pi _A / (1-\pi _A))$, where $\pi _A$ denotes the probability of a user having at least one accepted answer in a given year (see “Methods” section). We choose to do so—instead of modelling the acceptance rate of a user’s answers—because we find the user population to be approximately split between those who have at least one accepted answer and those who have none. The full results of the calibration of the above model, and the corresponding ROC curves, are shown in the Supplementary Information S1. Throughout the years, the model delivers excellent accuracy (with an AUC ranging between $76\%$ and $84\%$). The regression coefficients obtained for each covariate in each year of our analysis are illustrated in Fig. 4. For the first ten out of eleven years, specialisation ($H^A$) is found to be the leading contributor to a user’s ability to post high-quality answers, reaching its maximum relative importance in the early years of the platform, with some mild decline in more recent years. User activity (*n*) is the second main contributor, with an increasing trend suggesting that it may be overtaking specialisation (albeit the two coefficients are statistically indistinguishable both in 2018 and 2019). Notably, the number of tags *t* on which a user posts answers is the only covariate whose impact changes over time: before 2013-2014 it contributes positively to a user’s ability to post accepted answers, while it contributes negatively to it after then. This somewhat further strengthens the importance of specialisation, as it suggests that the more successful users are those who specialise on narrower sets of tags. The activity score *D* remains instead negatively correlated with the ability to post accepted answers throughout the entire time window of our analysis. Those results are robust with respect to changes in the threshold on the minimum number of posts for a user to be included in our analysis (see Supplementary Information S1).

We then build a multinomial logistic regression model to classify users (in each year) into three mutually exclusive categories: users whose posts have received zero votes, users whose posts have received only up-votes, and users whose posts have received both up- and down-votes. We neglect the case of users whose posts only receive down-votes, since in all years considered in our analysis they are less than $0.1\%$. We calibrate multinomial logistic regression models for the log-odds associated with the probability of belonging to the three above categories, using the aforementioned features as covariates. The full results are reported in the Supplementary Information S1, and show that no specific feature is systematically associated with a higher probability of attracting votes.

We then proceed to restrict our analysis to those users whose posts received at least one vote in a given year. To this end, we calibrate four stepwise regression models using as dependent variables the (logarithm of the) average number of up- or down-votes per post received by a user. Starting from a constant model, we use both forward and backward selection to select the best model (in terms of sum of squared residuals) based on the aforementioned covariates. With only few exceptions, the stepwise selection procedure results in a very simple model where the users’ activity—as quantified by their number of posts *n*—is the only statistically significant covariate. However, it is noteworthy that activity has a similar impact across board, both in terms of sign and magnitude. Namely, we find activity to have a negative impact on the number of votes received per post, both in the case of up- and down-votes and regardless of the type of post. Remarkably, in the case of questions such minimalistic models explain 60% or more of the variance. The full results of the calibration are reported in the Supplementary Information S1.

## Discussion

In this paper we presented a number of analyses aimed at understanding the relationship between specialisation and reputation in the domain of online decentralised platforms. Thanks to the lack of monetary incentives and its already long history, Stack Overflow represents an ideal environment to observe the development of such a relationship ‘in the wild’ over an extended period of time. Before we proceed with a critical discussion of our results, it is important to remind the reader that they are of a correlational nature, and therefore none of our analyses imply any causal link between Stack Overflow’s reputation system and the outcomes we report in this paper.

In line with previous studies on online platforms^21^, the 11 years of history covered in our analysis reveal how the Stack Overflow platform’s user base grew into a structured community, with different individuals taking on different roles. First, we documented how most of the platform’s user base quickly evolved into well defined supply and demand sides, represented by two large sub-communities of users characterized by their willingness to answer or pose questions, respectively. Second, we provided ample evidence on the emergence of specialisation at the level of topic selection in the users’ posts. Should the above findings be attributed to self-organisation or should they instead be interpreted as a direct response to the platform’s design and incentives? Plausibly, the very nature of Stack Overflow—a knowledge-sharing platform structured around questions and answers—is responsible for the emergence of sub-communities dedicated to posting answers and questions. Like other two-sided platforms, Stack Overflow naturally attracts users with markedly different needs (e.g., similarly to hosts and guests in accommodation platforms).

Specialisation with respect to topic selection is a more complicated phenomenon to unpack. We do not find it to be significantly correlated with the likelihood of attracting up- or down-votes to generic posts, suggesting that the quality of a user’s posts may be largely idiosyncratic (though we ought to acknowledge that user voting is known to be potentially affected by biases^29^). Conversely, we do find a statistically significant correlation between a user’s specialisation and the likelihood of their answers being accepted as the best one in response to a question. This is a notable asymmetry, as an equivalent selection mechanism is lacking in the case of questions, and no other user-generated feedback awards more reputation points on Stack Overflow than an accepted answer. We interpret these findings as a clear consequence of the incentives set in place by the platform’s reputation system, which naturally promote specialisation among users who answer questions as a strategy to secure high numbers of accepted answers and the reputational reward that ensues. Furthermore, providing high quality answers to posted questions is behaviourally consistent with theories of cooperation and its evolution, which demonstrate that helpful acts contribute to sustain cooperation in a social system even when not directly rewarded by their recipients^6–8^. In this respect, the competition to secure the reward associated to accepted answers represents a virtuous cycle that benefits the platform as a whole.

It is interesting to relate our results to findings about the users’ decision-making when choosing which answers to accept. Such decision-making has been found to be largely driven by heuristics, with selections being determined by factors such as the order in which answers appear or the amount of screen space they occupy^30^. It is therefore tempting to speculate that the selection process that takes place on posted answers may contribute to optimise user behaviour with respect to such heuristics.

Our findings illustrated in Fig. 4 shed light on the above point by identifying the salient traits of successful users. These are—on average—highly active and specialised users, whose specialisation progressively focuses on a narrower set of topics (as testified by the change in sign of the coefficient associated to the number of tags). Notably, these are not users who specialise in posting answers only, as their activity score *D* (see Eq. (1)) is negatively correlated with the likelihood of having answers accepted, suggesting that developing some expertise on both sides of a two-sided platform may unlock positive reputational spillovers.

Overall, our findings are in rather stark contrast with observations made in top-down environments (such as firms and corporations), where generalists are usually found to enjoy greater success than specialists^15–18^. Intuitively, this is likely due to the differences in uncertainty and stakes that players operating in different contexts need to face. For instance, the decision-making of the CEO of a large firm typically involves multiple tradeoffs and may be consequential to numerous people. Conversely, Stack Overflow is a low-stake environment for most users (with the likely exception of those who invest time in it in order to improve their career prospects, e.g., as developers^4^). In this respect, generalism and specialisation may be seen as strategic responses to the different types of complexity that actors in top-down vs bottom-up environments may need to deal with. In accordance with the law of requisite variety^31,32^, decision-makers in top-down environments need to be able to react to a huge variety of situations, which likely acts as a natural selection mechanism that favours generalist backgrounds. Users of decentralised platforms—such as Stack Overflow—are instead free to select the extent to which they want to interact with their environment, and to specialise accordingly.

We ought to acknowledge that the extent to which our findings may generalise to other decentralised online environments can only be the subject of speculation at this stage. Stack Overflow’s reputation system and the sustained success it has brought to the platform—with relatively minimal policy changes throughout the years—are rather unique. Other successful knowledge-sharing platforms have taken radically different approaches to foster trust within their user base. For instance, Wikipedia holds elections to promote reliable users to administrators. Similarly, comparisons with different reputation/feedback systems (e.g, textual reviews) are not straightforward. In this respect, the balance between specialisation and generalism in a platform’s user base may manifest itself in different ways depending on the platform’s incentives and design. However, we believe our work represents a first step towards the data-driven modelling of the relationship between specialisation and online reputation, and a blueprint that following studies may adapt to different environments and data sources.

## Methods

### Nestedness

In ecological systems, nestedness refers to a property typically observed in the networks describing species-species interactions. Let us assume that such interactions in a given system are represented by a weighted bipartite adjacency matrix *W*, whose entry $w_{ij}$ quantifies the strength of interaction between species *i* and *j*. In a perfectly nested matrix, an arrangement of rows and columns can be found such that the set of links in each row *i* (column *j*) contains the set of links in row $i+1$ (column $j+1$), and such that matrix entries satisfy $W_{ij} \le \min (W_{i-1,j},W_{i,j-1})$. It can be shown that among all possible connected bipartite networks with a fixed number of nodes and links, the one yielding the highest spectral radius $\rho (W)$ corresponds to a perfectly nested matrix^33^, where the spectral radius is defined as the largest singular value. Therefore, an ideal measure of nestedness in an empirical bipartite weighted matrix would be the ratio between its spectral ratio and that of the corresponding perfectly nested matrix with the same number of nodes and links. This, however, is unfeasible in practice due to the prohibitively high computational cost of identifying the perfectly nested matrix in the set via hard counting. Therefore, in our work we follow Staniczenko *et al*.^28^, and quantify the nestedness of a matrix with the *z*-score $z(\rho ) = (\rho (W) - {\overline{\rho }}(W))/\sigma (\rho (W))$, where ${\overline{\rho }}(W)$ and $\sigma (\rho (W))$ represent, respectively, the mean and standard deviation of the spectral radii computed over a sampled population of bipartite matrices with the same nodes and edges as *W*, but with randomly reshuffled link weights.

### Logistic regression model for user specialization

For each year in our analysis we calibrate the following logistic regression model:

3 $$\begin{aligned} \log \left( \frac{\pi _A}{1-\pi _A} \right) = \beta _0^{A} + \beta _{n}^{A} \log (n^A) + \beta _{t}^{A} \log (t^A) + \beta _H^{A} H^A + \beta _D^{A} D \ , \end{aligned}$$

where $\pi _A$ denotes the probability that at least one of the answers posted by a user (with at least 10 posts in the year under consideration) gets accepted, i.e., marked as the best one in response to a question. In the above expression *n* denotes the number of answers posted by a user, $t^A$ the number of tags associated with the corresponding questions, $H^A$ the specialization of the user as quantified by the Herfindahl index (see Eq. (2)), and *D* the user’s activity score (see Eq. (1)).

## Supplementary Information

Supplementary Information.

## Data Availability

The data used in this paper are freely accessible and can be downloaded via https://archive.org/details/stackexchange.
