# Supplementary material for: Quantifying the relationship between specialisation and reputation in an online platform
Source: Sci Rep. 2022 Oct 6;12:16699. doi: 10.1038/s41598-022-20767-7 (PMC9537143; doi:10.1038/s41598-022-20767-7)
Supplement: Supplementary file 1 — Supplementary Information. [file 41598_2022_20767_MOESM1_ESM.pdf]

Supplementary Information
Quantifying the relationship between specialisation and reputation in an online platform

Giacomo Livan,[1,2] Giuseppe Pappalardo,[3] Rosario N. Mantegna[4,5]

[1]*Department of Computer Science, University College London, London WC1E 6EA, United Kingdom*

[2]*Systemic Risk Centre, London School of Economics and Political Sciences, London WC2A 2AE, United Kingdom*

[3]*Dipartimento di Fisica 'Ettore Majorana', Università di Catania, Via S. Sofia, 64, 95123 Catania, Italy*

[4]*Dipartimento di Fisica e Chimica Emilio Segrè, Università di Palermo, Viale delle Scienze, Ed. 18, 90128, Palermo, Italy*

[5]*Complexity Science Hub Vienna, Josefstädter Strasse 39, 1080, Vienna, Austria*

Supplementary Tables

Supplementary Table 1. **Logistic regression model for the probability that a user's answer gets accepted**. We calibrate the model $\log(\pi_A/(1-\pi_A)) = \beta_0^A + \beta_n^A \log(n^A) + \beta_t^A \log(t^A) + \beta_H^A H^A + \beta_D^A D$, where π_A denotes the probability that a user has at least one accepted answer in a given year, n^A indicates the number of answers posted by a user, t^A the number of tags featured in the corresponding questions, H^A the user's Herfindahl index (Eq. (2) in the main paper), and D the user's activity score (Eq. (1) in the main paper). The three bottom rows report – respectively – the number N of users included in the model (i.e., users with at least 10 posts in the year of interest, of which at least one is an answer), the resulting regression model's area under the curve (AUC) and the model's χ^2 statistic. The corresponding ROC curves are shown in Supplementary Figure 3. Numbers in brackets indicate the standard errors of the estimated coefficients

	2009	2010	2011	2012	2013	2014	2015	2016	2017	2018	2019
β_0^A	-4.29***	-4.86***	4.79***	-4.66***	-4,39***	-4.10***	-4.15***	-4.10***	-3.93***	-3.91***	-3.77***
	(0.11)	(0.09)	(0.07)	(0.06)	(0.05)	(0.04)	(0.04)	(0.04)	(0.04)	(0.04)	(0.04)
β_n^A	1.95***	1.92***	2.00***	1.95***	1.90***	2.02***	2.02***	2.03***	2.07***	2.28***	2.44***
	(0.09)	(0.06)	(0.05)	(0.04)	(0.03)	(0.03)	(0.03)	(0.03)	(0.03)	(0.03)	(0.03)
β_t^A	0.00	0.34***	0.25***	0.22***	0.07*	-0.18***	-0.20***	-0.25***	-0.38***	-0.64***	-0.91***
	(0.11)	(0.08)	(0.07)	(0.05)	(0.04)	(0.04)	(0.04)	(0.04)	(0.04)	(0.04)	(0.04)
β_H^A	2.98***	3.67***	3.45***	3.37***	3.04***	2.73***	2.76***	2.81***	2.59***	2.48***	2.23***
	(0.19)	(0.14)	(0.11)	(0.09)	(0.07)	(0.07)	(0.07)	(0.07)	(0.07)	(0.07)	(0.07)
β_D^A	-0.75***	-0.94***	-0.96***	-0.96***	-0.89***	-0.81***	-0.78***	-0.74***	-0.77***	-0.80***	-0.77***
	(0.03)	(0.03)	0.02	(0.02)	(0.01)	(0.12)	(0.02)	(0.01)	(0.02)	(0.01)	(0.01)
N	33,669	60,035	100,568	151,132	198,553	221,941	237,108	241,120	232,722	207,945	199,795
AUC	0.84	0.83	0.83	0.82	0.80	0.79	0.78	0.78	0.77	0.76	0.76
χ^2 statistics ($\times 10^4$)	1.01***	1.85***	3.07***	4.36***	5.52**	5.87***	6.31***	6.23***	5.69***	5.10***	4.96***

$^{***}p < 0.01$, $^{**}p < 0.05$, $^{*}p < 0.1$

Supplementary Table 2. **Multinomial logistic regression model for the probability that a user's answers attract votes.** We calibrate the model $\log(\pi^{(u,A}/\pi^{(z,A)}) = \beta_0^{(u,A)} + \beta_n^{(u,A)}\log(n^A) + \beta_t^{(u,A)}\log(t^A) + \beta_H^{(u,A)}H^A + \beta_D^{(u,A)}D$; $\log(\pi^{(u,A}/\pi^{(z,A)}) = \beta_0^{(v,A)} + \beta_n^{(v,A)}\log(n^A) + \beta_t^{(v,A)}\log(t^A) + \beta_H^{(v,A)}H^A + \beta_D^{(v,A)}D$, where $\pi^{(z,A)}$, $\pi^{(u,A)}$ and $\pi^{(v,A)}$ indicate – respectively – the probability that a user's posted answers receive zero votes, only up-votes, and both up- and down-votes in a given year. n^A indicates the number of answers posted by a user, t^A the number of tags featured in the corresponding questions, H^A the user's Herfindahl index (Eq. (2) in the main paper), and D the user's activity score (Eq. (1) in the main paper). The four bottom rows report the total number of users (N), and the fractions of users whose posted answers received only up-votes (N_u), both up- and down-votes (N_v) and zero votes (N_z). Numbers in brackets indicate the standard errors of the estimated coefficients.

	2009	2010	2011	2012	2013	2014	2015	2016	2017	2018	2019
$\beta_0^{(u,A)}$	-2.43***	-2.54***	-2.64***	-2.72***	-2.84***	-2.99**	-3.12***	-3.35***	-3.51***	-3.79***	-3.78***
	(0.09)	(0.07)	(0.06)	(0.06)	(0.05)	(0.06)	(0.06)	(0.06)	(0.07)	(0.08)	(0.10)
$\beta_n^{(u,A)}$	-0.05	-0.11	-0.12**	-0.03	-0.03	0.00	0.02	-0.16***	-0.04	-0.06	0.00
	(0.10)	(0.07)	(0.06)	(0.05)	(0.04)	(0.04)	(0.04)	(0.05)	(0.05)	(0.06)	(0.07)
$\beta_t^{(u,A)}$	0.24*	0.26***	0.27***	0.12*	0.12**	0.08	0.05	0.26***	0.12*	0.18**	0.02
	(0.12)	(0.09)	(0.08)	(0.06)	(0.06)	(0.06)	(0.06)	(0.07)	(0.07)	(0.08)	(0.09)
$\beta_H^{(u,A)}$	0.40*	0.44***	0.09	0.19*	0.10	0.08	0.04	0.24*	0.15	0.45***	0.13
	(0.22)	(0.16)	(0.14)	(0.12)	(0.11)	(0.12)	(0.12)	(0.13)	(0.15)	(0.16)	(0.18)
$\beta_D^{(u,A)}$	-0.10**	0.06*	-0.03	0.03	-0.01	0.00	0.00	0.06***	0.00	0.02	0.06*
	(0.04)	(0.03)	(0.02)	(0.02)	(0.02)	(0.02)	(0.02)	(0.02)	(0.03)	(0.03)	(0.03)
$\beta_0^{(v,A)}$	-2.15***	-2.97***	-3.28***	-3.45***	-3.49***	-4.01***	-3.98***	-4.47***	-4.73***	-5.12***	-5.61***
	(0.09)	(0.08)	(0.07)	(0.08)	(0.08)	(0.09)	(0.10)	(0.08)	(0.13)	(0.16)	(0.20)
$\beta_n^{(v,A)}$	0.07	-0.09	-0.14*	0.00	0.05	-0.04	0.14*	-0.01	-0.09	0.36***	-0.07
	(0.09)	(0.08)	(0.07)	(0.07)	(0.06)	(0.07)	(0.07)	(0.08)	(0.09)	(0.11)	(0.14)
$\beta_t^{(v,A)}$	0.10	0.31***	0..34***	0.13	-0.04	0.16*	-0.14	0.08	0.16	0.29**	0.18
	(0.10)	(0.10)	(0.09)	(0.09)	(0.09)	(0.09)	(0.10)	(0.11)	(0.13)	(0.15)	(0.20)
$\beta_H^{(v,A)}$	-0.20	0.55***	0.51***	0.19	0.02	0.08	-0.33	0.27	0.40	0.20	0.68*
	(0.22)	(0.18)	(0.16)	(0.16)	(0.16)	(0.19)	(0.21)	(0.22)	(0.25)	(0.31)	(0.36)
$\beta_D^{(v,A)}$	-0.11***	0.00	-0.03	-0.03	0.04	-0.03	-0.07**	0.03	0.03	-0.15*	0.06
	(0.04)	(0.03)	(0.03)	(0.03)	(0.03)	(0.03)	(0.03)	(0.04)	(0.05)	(0.06)	(0.07)
N	33,669	60,035	100,573	151,132	198,553	221,941	237,108	241,120	232,722	207,950	199,795
$N_u(\%)$	11.3 %	10.1%	8.69%	7.48%	6.48%	5.57%	4.89%	4.21%	3.51%	2.97%	2.41%
$N_v(\%)$	13.7%	7.90%	5.59%	3.94%	2.91%	2.15%	1.72%	1.32%	1.05%	0.76%	0.51%
$N_z(\%)$	74.9%	81.9%	85.6%	88.5%	90.5%	92.2%	93.3%	94.3%	95.4%	96.2%	97.0%

$^{***}p < 0.01$, $^{**}p < 0.05$, $^{*}p < 0.1$

Supplementary Table 3. **Multinomial logistic regression model for the probability that a user's questions attract votes.** We calibrate the model $\log(\pi^{(u,Q}/\pi^{(z,Q)}) = \beta_0^{(u,Q)} + \beta_n^{(u,Q)}\log(n^Q) + \beta_t^{(u,Q)}\log(t^Q) + \beta_H^{(u,Q)}H^Q + \beta_D^{(u,Q)}D$; $\log(\pi^{(u,Q}/\pi^{(z,Q)}) = \beta_0^{(v,Q)} + \beta_n^{(v,Q)}\log(n^Q) + \beta_t^{(v,Q)}\log(t^Q) + \beta_H^{(v,Q)}H^Q + \beta_D^{(v,Q)}D$, where $\pi^{(z,Q)}$, $\pi^{(u,Q)}$ and $\pi^{(v,Q)}$ indicate – respectively – the probability that a user's posted questions receive zero votes, only up-votes, and both up- and down-votes in a given year. n^Q indicates the number of questions posted by a user, t^Q the number of tags featured in the such questions, H^Q the user's Herfindahl index (Eq. (2) in the main paper), and D the user's activity score (Eq. (1) in the main paper). The four bottom rows report the total number of users (N), and the fractions of users whose posted quesions received only up-votes (N_u), both up- and down-votes (N_v) and zero votes (N_z). Numbers in brackets indicate the standard errors of the estimated coefficients.

	2009	2010	2011	2012	2013	2014	2015	2016	2017	2018	2019
$\beta_0^{(u,Q)}$	-1.88***	-1.85***	-2.38***	-2.53***	-2.71***	-3.02***	-3.02***	-3.37***	-3.58***	-3.72***	-3.89***
	(0.11)	(0.08)	(0.07)	(0.06)	(0.06)	(0.12)	(0.08)	(0.08)	(0.10)	(0.11)	(0.13)
$\beta_n^{(u,Q)}$	0.00	0.11**	0.01	0.04	-0.08**	-0.05	0.01	-0.05	-0.02	-0.07	-0.06
	(0.07)	(0.05)	(0.04)	(0.03)	(0.03)	(0.04)	(0.04)	(0.04)	(0.05)	(0.05)	(0.06)
$\beta_t^{(u,Q)}$	0.15	-0.08	0.05	0.00	0.12**	0.09*	-0.03	0.10*	0.06	0.10	0.00
	(0.10)	(0.07)	(0.06)	(0.05)	(0.05)	(0.05)	(0.05)	(0.06)	(0.07)	(0.08)	(0.09)
$\beta_H^{(u,Q)}$	-0.20	0.20	0.00	0.09	0.01	0.29**	0.00	0.19	0.02	0.08	0.09
	(0.22)	(0.16)	(0.14)	(0.13)	(0.13)	(0.13)	(0.14)	(0.15)	(0.18)	(0.21)	(0.23)
$\beta_D^{(u,Q)}$	0.09***	0.08***	0.04**	0.03	0.07***	0.01	0.03	0.03	0.03	0.05*	-0.03
	(0.03)	(0.02)	(0.02)	(0.02)	(0.02)	(0.02)	(0.02)	(0.02)	(0.02)	(0.03)	(0.03)
$\beta_0^{(v,Q)}$	-2.90***	-3.07***	-3.09***	-3.37***	-3.60***	-3.60***	-3.82***	-4.19***	-4.29***	-4.77***	-4.85***
	(0.15)	(0.14)	(0.13)	(0.11)	(0.10)	(0.12)	(0.12)	(0.14)	(0.16)	(0.18)	(0.21)
$\beta_n^{(v,Q)}$	-0.07	0.02	0.14**	0.00	0.00	0.01	0.03	0.02	0.03	0.06	0.00
	(0.11)	(0.09)	(0.07)	(0.06)	(0.05)	(0.06)	(0.06)	(0.07)	(0.07)	(0.09)	(0.10)
$\beta_t^{(v,Q)}$	0.27*	-0.03	-0.26***	-0.07	-0.09	-0.17*	-0.15*	-0.08	-0.13	-0.04	0.00
	(0.14)	(0.12)	(0.10)	(0.08)	(0.08)	(0.09)	(0.09)	(0.10)	(0.11)	(0.13)	(0.14)
$\beta_H^{(v,Q)}$	0.29	0.38	-0.36	0.07	0.22	-0.30	-0.12	0.02	-0.16	0.08	-0.31
	(0.30)	(0.27)	(0.25)	(0.21)	(0.19)	(0.23)	(0.23)	(0.25)	(0.29)	(0.34)	(0.40)
$\beta_D^{(v,Q)}$	0.07*	0.01	0.02	-0.02	-0.02	-0.03	0.02	-0.01	0.00	0.06	0.09
	(0.04)	(0.04)	(0.03)	(0.03)	(0.03)	(0.03)	(0.03)	(0.04)	(0.04)	(0.05)	(0.05)
N	30,421	59,241	104,794	158,229	209,674	234,670	246,786	246,786	240,697	213,597	214,424
$N_u(\%)$	16.3 %	14.5%	10.6%	8.01%	6.41%	5.11%	4.41%	3.62%	2.92%	2.43%	1.82%
$N_v(\%)$	7.03%	3.96%	2.92%	2.64%	2.19%	1.72%	1.57%	1.28%	1.05%	0.87%	0.69%
$N_z(\%)$	76.7%	81.5%	86.5%	89.3%	91.3%	93.0%	93.9%	95.0%	95.9%	96.6%	97.4%

$^{***}p < 0.01$, $^{**}p < 0.05$, $^{*}p < 0.1$

Supplementary Table 4. **Stepwise linear regression model for the number of up-votes received by a user's answers**. We calibrate the model $\log(v^{\uparrow A}) = \beta_0^{\uparrow A} + \beta_n^{\uparrow A} \log(n^A) + \beta_t^{\uparrow A} \log(t^A) + \beta_H^{\uparrow A} H^A + \beta_D^{\uparrow A} D$, where $v^{\uparrow A}$ denotes the number of up-votes received by a user's posted answers, n^A indicates the number of answers posted by a user, t^A the number of tags featured in the corresponding questions, H^A the user's Herfindahl index (Eq. (2) in the main paper), and D the user's activity score (Eq. (1) in the main paper). The three bottom rows report – respectively – the number N of users included in the model (i.e., users with at least 10 posts in the year of interest and with at least one up-vote to their posted answers), the resulting regression model's R^2 coefficient and the model's F statistic. Numbers in brackets indicate the standard errors of the estimated coefficients.

**Variable**	2009	2010	2011	2012	2013	2014	2015	2016	2017	2018	2019
$\beta_0^{\uparrow A}$	2.96***	2.61***	1.90***	2.35***	2.37***	2.03***	1.96***	1.88***	1.69***	1.75***	1.27***
	(0.06)	(0.04)	(0.20)	(0.04)	(0.04)	(0.03)	(0.03)	(0.03)	(0.03)	(0.07)	(0.04)
$\beta_n^{\uparrow A}$	-0.91***	-0.91***	-1.07***	-1.08***	-1.01***	-0.96***	-0.98***	-0.98***	-0.98***	-0.93***	-0.98***
	(0.02)	(0.01)	(0.08)	(0.06)	(0.01)	(0.01)	(0.01)	(0.01)	(0.01)	(0.07)	(0.02)
$\beta_t^{\uparrow A}$	-	-	0.44***	0.17**	-	-	-	-	-	-0.20**	-
			(0.13)	(0.08)						(0.08)	
$\beta_H^{\uparrow A}$	-	-	1.22***	-	-	-	-	-	-	-	-
			(0.01)							-	
$\beta_D^{\uparrow A}$	-	-	-	-	0.08***	-	-	-	-	-	-
					(0.03)						
N	8,423	10,787	14,364	17,257	18,643	17,134	15,683	13,318	10,610	7,775	5,842
R^2	0.28	0.31	0.33	0.33	0.35	0.34	0.35	0.36	0.38	0.41	0.42
F statistic ($\times 10^3$)	3.35***	4.90***	1.78***	4.22***	4.96***	8.83***	8.56***	7.49***	6.36***	1.79***	4.18***

$^{***}p < 0.01$, $^{**}p < 0.05$, $^{*}p < 0.1$

Supplementary Table 5. **Stepwise linear regression model for the number of down-votes received by a user's answers**. We calibrate the model $\log(v^{\downarrow A}) = \beta_0^{\downarrow A} + \beta_n^{\downarrow A} \log(n^A) + \beta_t^{\downarrow A} \log(t^A) + \beta_H^{\downarrow A} H^A + \beta_D^{\downarrow A} D$, where $v^{\downarrow A}$ denotes the number of down-votes received by a user's posted answers, n^A indicates the number of answers posted by a user, t^A the number of tags featured in the corresponding questions, H^A the user's Herfindahl index (Eq. (2) in the main paper), and D the user's activity score (Eq. (1) in the main paper). The three bottom rows report – respectively – the number N of users included in the model (i.e., users with at least 10 posts in the year of interest and with at least one down-vote to their posted answers), the resulting regression model's R^2 coefficient and the model's F statistic. Numbers in brackets indicate the standard errors of the estimated coefficients.

**Variable**	2009	2010	2011	2012	2013	2014	2015	2016	2017	2018	2019
$\beta_0^{\downarrow A}$	1.27***	1.03***	0.90***	0.93***	0.72***	0.61***	0.58***	0.59***	0.49***	0.45***	0.36***
	(0.05)	(0.04)	(0.06)	(0.05)	(0.03)	(0.03)	(0.03)	(0.03)	(0.04)	(0.04)	(0.07)
$\beta_n^{\downarrow A}$	-0.97***	-0.97***	-0.98***	-1.02***	-1.00***	-0.99***	-1.00***	-1.01***	-0.98***	-0.98***	-0.93***
	(0.01)	(0.01)	(0.01)	(0.01)	(0.01)	(0.01)	(0.01)	(0.01)	(0.01)	(0.01)	(0.03)
$\beta_t^{\downarrow A}$	-	-	-	-	-	-	-	-	-	-	-
$\beta_H^{\downarrow A}$	-	-	0.27**	-0.27**	-	-	-	-	-		-
			(0.13)	(0.12)							
$\beta_D^{\downarrow A}$	-	-	-	-		-	-	-	-	-	-0.07**
											(0.03)
N	4,659	4,816	5,721	6,080	5,937	4,973	4,302	3,379	2,606	1,755	1,131
R^2	0.55	0.62	0.64	0.67	0.67	0.68	0.69	0.69	0.69	0.71	0.71
F statistisc ($\times 10^3$)	5.58***	7.78***	5.03***	6.25***	10.6***	10.6***	9.70***	7.43***	5.80***	2.72***	0.94***

$^{***}p < 0.01$, $^{**}p < 0.05$, $^{*}p < 0.1$

Supplementary Table 6. **Stepwise linear regression model for the number of up-votes received by a user's questions**. We calibrate the model $\log(v^{\uparrow Q}) = \beta_0^{\uparrow Q} + \beta_n^{\uparrow Q} \log(n^Q) + \beta_t^{\uparrow Q} \log(t^Q) + \beta_H^{\uparrow Q} H^Q + \beta_D^{\uparrow Q} D$, where $v^{\uparrow Q}$ denotes the number of up-votes received by a user's posted answers, n^Q indicates the number of answers posted by a user, t^Q the number of tags featured in the corresponding questions, H^Q the user's Herfindahl index (Eq. (2) in the main paper), and D the user's activity score (Eq. (1) in the main paper). The three bottom rows report – respectively – the number N of users included in the model (i.e., users with at least 10 posts in the year of interest and with at least one up-vote to their posted questions), the resulting regression model's R^2 coefficient and the model's F statistic. Numbers in brackets indicate the standard errors of the estimated coefficients.

**Variable**	2009	2010	2011	2012	2013	2014	2015	2016	2017	2018	2019
$\beta_0^{\uparrow Q}$	2.72***	2.28***	2.28***	2.11***	1.80***	1.47***	1.49***	1.39***	1.28***	1.01***	0.86***
	(0.06)	(0.05)	(0.04)	(0.06)	(0.03)	(0.03)	(0.03)	(0.03)	(0.04)	(0.04)	(0.04)
$\beta_n^{\uparrow Q}$	-0.89***	-1.17***	-1.08***	-1.13***	-0.98***	-1.01***	-0.97***	-0.96***	-0.98***	-0.95***	-0.96***
	(0.02)	(0.07)	(0.04)	(0.04)	(0.01)	(0.03)	(0.01)	(0.01)	(0.01)	(0.01)	(0.02)
$\beta_t^{\uparrow Q}$	-	0.28***	0.11**	-	-	0.08**	-	-	-	-	-
		(0.07)	(0.05)			(0.04)					
$\beta_H^{\uparrow Q}$	-	-	-	-	-	-	-	-	-	-	-
$\beta_D^{\uparrow Q}$	0.12	-	-	-		-	0.05**	-	-	-	-
	(0.04)						(0.02)				
N	7,089	10,923	14,129	16,850	18,033	16,041	14,763	12,175	9,564	7,049	5,377
R^2	0.23	0.26	0.29	0.30	0.31	0.30	0.32	0.33	0.35	0.37	0.41
F statistisc ($\times 10^3$)	1.05***	1.90***	2.92***	2.42***	8.07***	3.48***	3.52***	5.95***	5.12***	3.71***	3.71***

$^{***}p < 0.01$, $^{**}p < 0.05$, $^{*}p < 0.1$

Supplementary Table 7. **Stepwise linear regression model for the number of down-votes received by a user's questions**. We calibrate the model $\log(v^{\downarrow Q}) = \beta_0^{\downarrow Q} + \beta_n^{\downarrow Q}\log(n^Q) + \beta_t^{\downarrow Q}\log(t^Q) + \beta_H^{\downarrow Q} H^Q + \beta_D^{\downarrow Q} D$, where $v^{\downarrow Q}$ denotes the number of down-votes received by a user's posted answers, n^Q indicates the number of answers posted by a user, t^Q the number of tags featured in the corresponding questions, H^Q the user's Herfindahl index (Eq. (2) in the main paper), and D the user's activity score (Eq. (1) in the main paper). The three bottom rows report – respectively – the number N of users included in the model (i.e., users with at least 10 posts in the year of interest and with at least one down-vote to their posted questions), the resulting regression model's R^2 coefficient and the model's F statistic. Numbers in brackets indicate the standard errors of the estimated coefficients.

**Variable**	2009	2010	2011	2012	2013	2014	2015	2016	2017	2018	2019
$\beta_0^{\downarrow Q}$	0.68***	0.61***	0.62***	0.66***	0.66***	0.59***	0.66***	0.54***	0.54***	0.54***	0.44***
	(0.05)	(0.04)	(0.04)	(0.03)	(0.03)	(0.03)	(0.04)	(0.04)	(0.04)	(0.05)	(0.05)
$\beta_n^{\downarrow Q}$	-0.98***	1.02***	-1.03***	-1.03***	-1.02***	-1.01***	-1.03***	-0.99***	-1.00***	-1.01***	-0.97***
	(0.02)	(0.02)	(0.01)	(0.01)	(0.01)	(0.01)	(0.01)	(0.01)	(0.02)	(0.02)	(0.02)
$\beta_t^{\downarrow Q}$	-	-	-	-	-	-	-	-	-	-	-
$\beta_H^{\downarrow Q}$	-	-	-	-	-	-	-	-	-		-
$\beta_D^{\downarrow Q}$	-	-	-	-		-	-	-	-	-	
N	2,144	2.387	3,130	4,300	4,831	4,358	4,179	3,485	2,830	2,079	1,718
R^2	0.62	0.65	0.66	0.63	0.62	0.61	0.59	0.58	0.58	0.61	0.58
F statistisc ($\times 10^3$)	3.50***	4.51***	6.08***	7.84***	7.84***	6.83***	6.11***	3.89***	3.89***	3.21***	2.34***

$^{***}p < 0.01$, $^{**}p < 0.05$, $^{*}p < 0.1$

Supplementary Figures

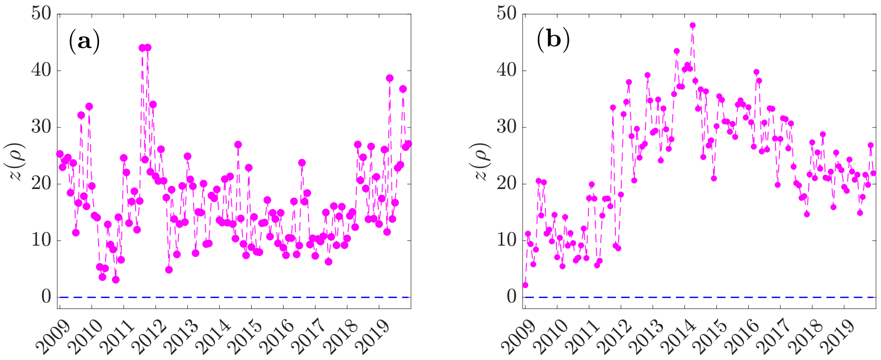

Supplementary Figure 1. **Evidence of nestedness in Stack Overflow's user-tag bipartite networks.** **a** z-scores of the spectral radius ρ calculated in the monthly user-tag networks of A-users (i.e., users with activity score $D = 1$, see Eq. (1) in the main paper). **b** Same quantity calculated in the monthly user networks of Q-users ($D = -1$). In both panels, z-scores are calculated with the procedure put forward in Staniczenko et al., *Nature Communications* **4**, 1-6 (2013).

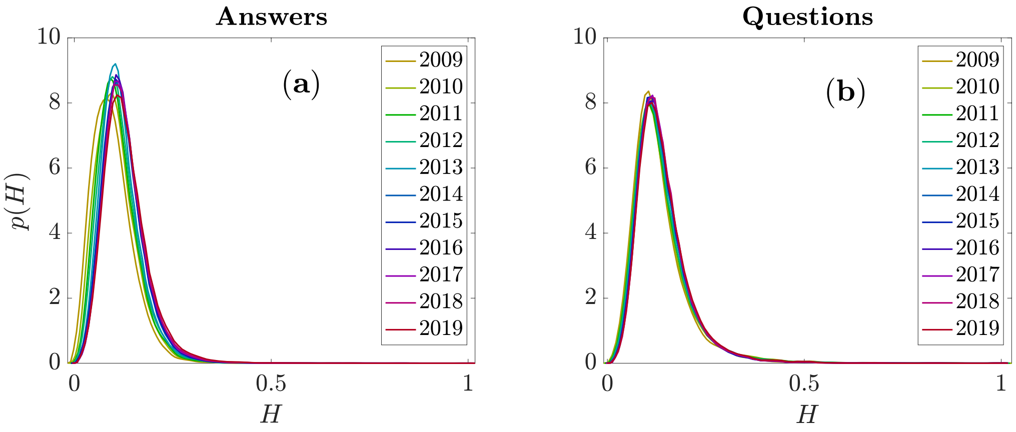

Supplementary Figure 2. **Empirical density of the Herfindahl index.** **a** Annual distribution of user specialisation with respect to tags in the case of answers. **b** Annual distribution of user specialisation with respect to tags in the case of questions.

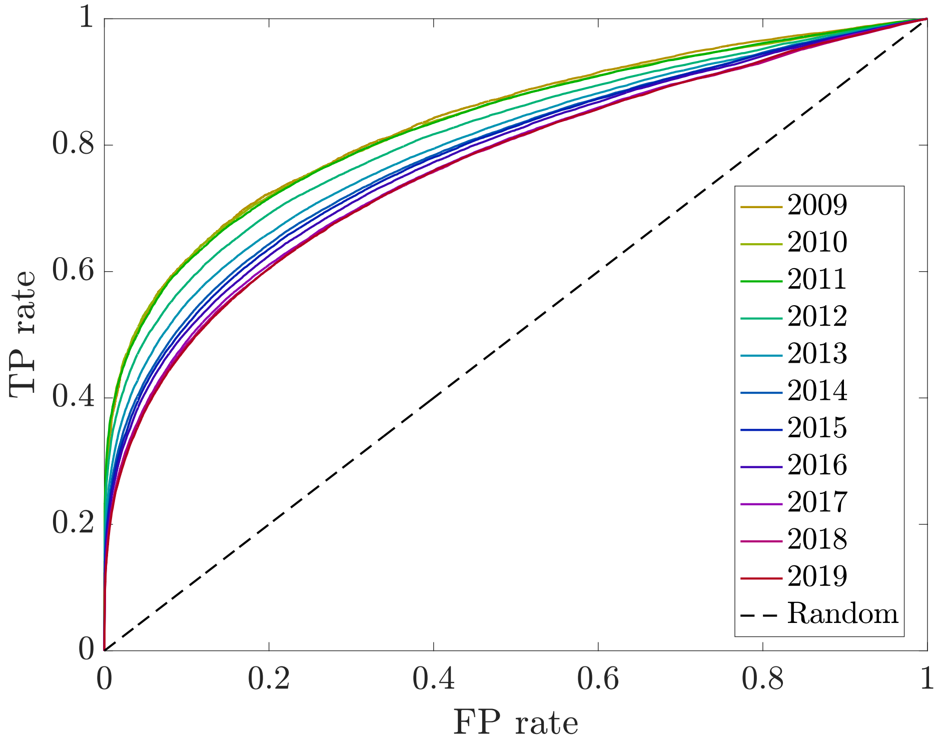

Supplementary Figure 3. **Model performance of the logistic regressions for accepted answers**. ROC curves for the models in Supplementary Table 1.

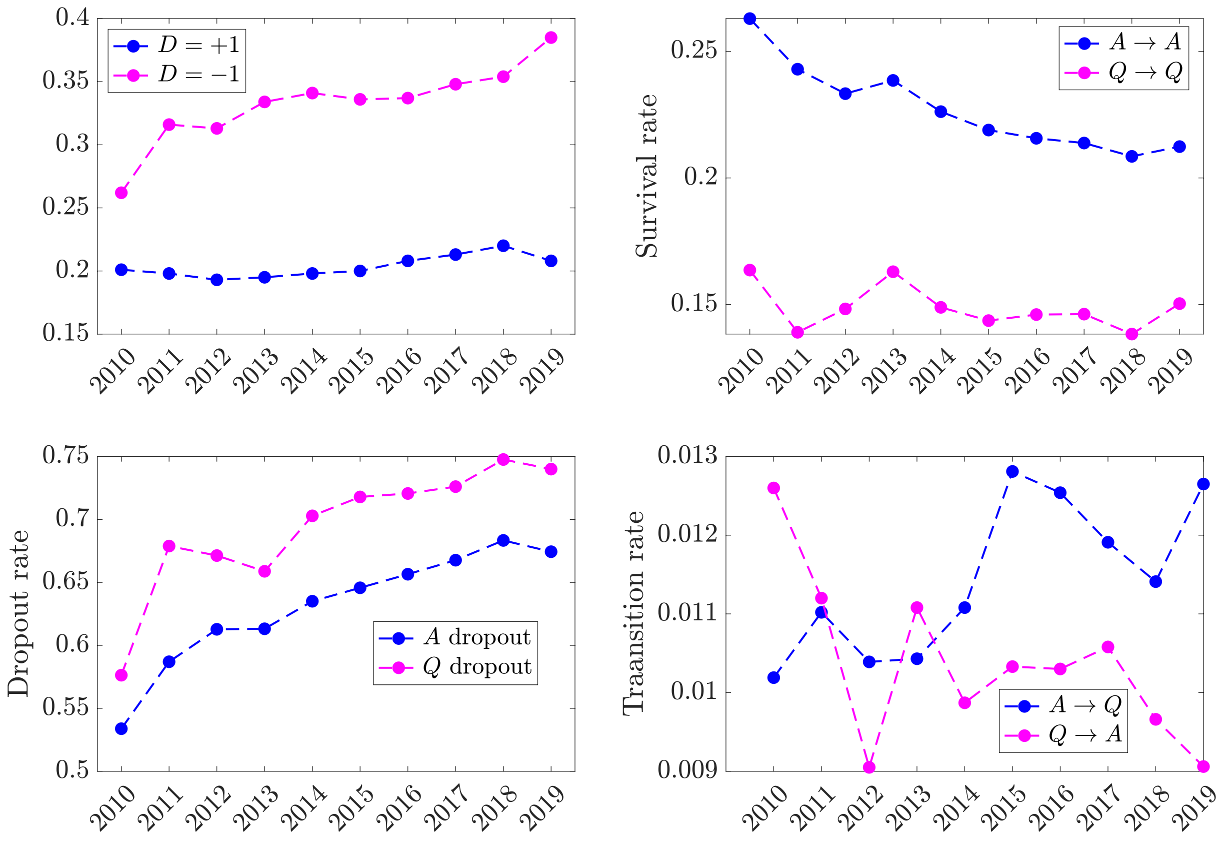

Supplementary Figure 4. **Characterisation of Stack Overflow's user base with a threshold on the minimum number of posts by a user in a year set to 5**. The panels in the figure are the equivalent to those in Fig. 2 of the main paper (where we use a threshold of 10). Top left: Annual percentage of A- and Q-users. Top right: Annual survival probabilities for A- (blue) and Q-users (magenta), defined as the empirically estimated probabilities for users belonging to either group to belong to the same group in the following year. Bottom left: Annual dropout rates for A- (blue) and Q-users (magenta), defined as the empirically estimated probabilities for users belonging to either group to either leave the platform or fall below the minimum threshold of 10 posts per year to be considered in our analysis. Bottom right: Annual transition rates from A- to Q-users (blue) and vice versa (magenta), defined as the empirically estimated probabilities for users belonging to one group to transition to the other one the following year.

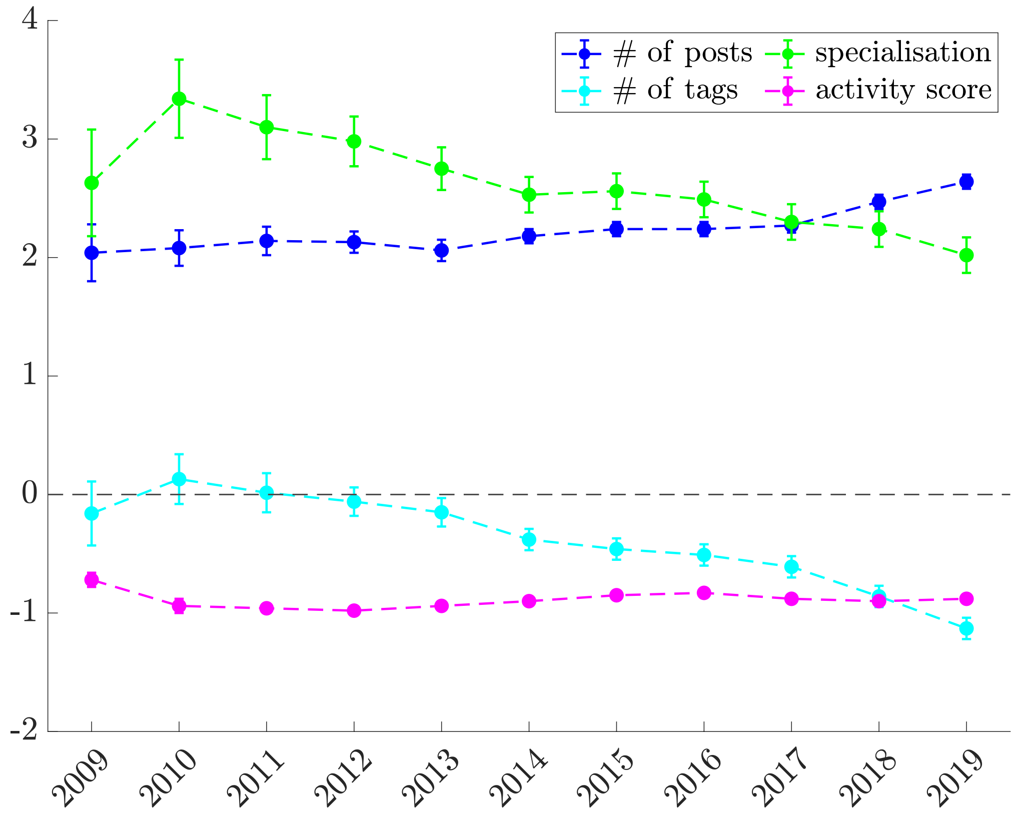

Supplementary Figure 5. **Logistic regression results for the probability that a user has at least one accepted answer in a given year (with threshold on the minimum number of posts by a user in a year set to 5)**. Dots represent the values of the regression coefficients estimated for the four covariates included in the model, shown in the legend. Error bars show the standard errors on the coefficients times three
